# The Mental Models Training App: Enhancing verbal reasoning through a cognitive training mobile application

**DOI:** 10.3389/fpsyg.2023.1150210

**Published:** 2023-03-10

**Authors:** Robert A. Cortes, Adam B. Weinberger, Adam E. Green

**Affiliations:** ^1^Department of Psychology, Georgetown University, Washington, DC, United States; ^2^Interdisciplinary Program in Neuroscience, Georgetown University, Washington, DC, United States

**Keywords:** mental models, cognitive training, reasoning, translational research, cognitive enhancement, mobile application

## Abstract

**Introduction:**

Reasoning is a complex form of human cognition whose nature has long been debated. While a number of neurocognitive mechanisms for deductive reasoning have been offered, one of the most prominent accounts is Mental Model Theory (MMT). According to MMT, humans are able to manipulate and represent information for reasoning and problem solving by leveraging the brain’s evolved visuospatial resources. Thus, when solving deductive reasoning problems, reasoners build “mental models” of the essential pieces of information conveyed in the premises, with their relations to each other represented spatially—even when the information contained within a reasoning problem is not intrinsically spatial. Crucially, taking a spatially-based approach, such as building mental models, supports higher accuracy on deductive reasoning problems. However, no study has empirically tested whether explicitly training this mental modeling ability leads to improved deductive reasoning performance.

**Method:**

Therefore, we designed the Mental Models Training App, a cognitive training mobile application which requires participants to complete increasingly difficult reasoning problems while using an external mental modeling tool. In this preregistered study (https://osf.io/4b7kn), we conducted a between-subjects experiment (*N* = 301) which compared the Mental Models Training App to 3 distinct control conditions in order to examine which specific components (if any) of the training were causally responsible for improved reasoning performance.

**Results:**

Results demonstrate that, when compared to a passive control condition, the Mental Models Training App led to improvements in adults’ verbal deductive reasoning performance both during and after the training intervention. However, contrary to our preregistered hypotheses, the training-induced improvements were not significantly larger than the effects of the active control conditions—one which included adaptive practice of the reasoning problems, and one which included adaptive practice as well as a spatial alphabetization control task.

**Discussion:**

Therefore, while the present results demonstrate the ability of the Mental Models Training App to enhance verbal deductive reasoning, they do not support the hypothesis that directly training participants mental modeling ability yields improved performance beyond the effects of adaptive practice of reasoning. Future research should examine the long-term effects of repeated usage of the Mental Models Training App, as well as transfer effects to other forms of reasoning. Finally, we present the Mental Models Training App as a free mobile application available on the Apple App store (https://apps.apple.com/us/app/mental-models-training/id1664939931), in the hope that this translational research may be utilized by the general public to improve their reasoning ability.

## 1. Introduction

Complex human thinking and reasoning is a recent evolutionary arrival. The primate brain evolved to interact with objects in space rather than interact with complex logic structures, so a great deal of the cerebral cortex is devoted to visuospatial and motor processing (Byrne and Johnson-Laird, 1989; Waltz et al., 1999; Byrne et al., 2007; Kravitz et al., 2011). According to a prominent account in cognitive science—mental model theory (MMT)—human reasoning and problem-solving co-opts previously evolved neural machinery for visuospatial and motor processing to internally represent and manipulate information (Johnson-Laird, 1980, 2010; Tversky, 1991; Wai et al., 2009). In other words, people form internal, spatially arranged “mental models” of relevant information, suggesting a connection between mental modeling ability and spatial cognition (e.g., related pieces of information are close together in space and unrelated pieces of information are far apart). Consistent with this perspective, emerging work indicates that spatial cognition is a malleable neurocognitive resource that supports deductive verbal reasoning (Collins and Gentner, 1987; Johnson-Laird, 2010; Uttal et al., 2013a,b; Cortes et al., 2022). The well-established role of mental modeling as a form of spatial cognition that supports verbal reasoning suggests that, if mental modeling can be trained through explicit spatialization of information, verbal reasoning performance can be enhanced. The goal of the present study was to train mental modeling using a mobile application and test for improvements in verbal deductive reasoning performance.

Mental model theory has been highly influential in the cognitive and brain sciences for several decades (Johnson-Laird, 1980; Byrne and Johnson-Laird, 1989; Goodwin and Johnson-Laird, 2005), and this literature has described mental modeling as a resource that generalizes across multiple forms of reasoning. Deductive verbal reasoning, for example, is supported by the formation and manipulation of mental models (Knauff and Johnson-Laird, 2002; Goodwin and Johnson-Laird, 2005; Knauff, 2009). In a deductive verbal reasoning problem, one must deduce whether a conclusion logically follows from premises (e.g., Premise 1: The dog is better than the cat/Premise 2: The cat is better than the frog/Conclusion: The dog is better than the frog). In such an example, a reasoner might represent the better option as *above* a worse option, “spatializing” the concept of goodness, which is not inherently spatial. Several theories of human reasoning suggest that these sorts of problems, often called linear syllogisms, are solved using internal representations which are spatially ordered (De Soto et al., 1965; Huttenlocher, 1968; Byrne and Johnson-Laird, 1989; Khemlani and Johnson-Laird, 2012; Ragni and Knauff, 2013). Notably, the extent to which reasoners are able to apply such mental models is associated with variability in task performance; building superior mental models has been associated with higher accuracy on deductive reasoning tasks (Galotti et al., 1986; Roberts, 2000; Schaeken et al., 2014). However, no study has empirically tested whether it is possible to explicitly training this mental modeling ability.

Although mental model training is thus-far untested, there is reason to believe that mental modeling can be improved through targeted interventions. For instance, many other visuospatial and motor cognitive resources are trainable and show transfer to untrained reasoning tasks (Adkins et al., 2006; Forgeard et al., 2008; Sanchez, 2012; Frick and Möhring, 2016; Lowrie et al., 2017). Educational psychology has also shown promise for training spatial cognition, which is thought to support mental modeling during reasoning (Byrne and Johnson-Laird, 1989; Johnson-Laird, 2004; Knauff, 2009). Meta-analytic evidence indicates that training on a range of spatial tasks leads to improvement on the trained abilities and may yield transfer to untrained STEM-related tasks (Uttal et al., 2013a). Emerging research has highlighted neural and behavioral changes during verbal reasoning following participation in spatially focused curricula in real-world classroom (Cortes et al., 2022). While encouraging, other spatial training studies have failed to produce lasting transfer (Mix and Cheng, 2012; Xu and LeFevre, 2016). Notably, none of this work has tested whether it is possible to directly train the mental modeling resource itself, and whether this would lead to improved verbal deductive reasoning performance.

Training efforts to improve spatial thinking reflect a growing emphasis within psychology and neuroscience to use cognitive training programs to improve general cognitive ability (CGA; Sala and Gobet, 2019). Generally, these training paradigms follow a similar logic: If Tasks X, Y, and Z require Cognitive Skill A—and Cognitive Skill A influence GCA—then training on Tasks X, Y, and/or Z can transfer to improve GCA. In other words, enhancing a domain-general cognitive ability is be achieved by a domain-specific training (Taatgen, 2021).

Most of these cognitive training efforts have focused on working memory (Jaeggi et al., 2008; Shipstead et al., 2012a,b). This is not surprising given the extensive literature demonstrating the strong positive relationship between working memory and a range of cognitive abilities (e.g., executive function, fluid intelligence, verbal reasoning, and mathematical achievement; Daneman and Carpenter, 1980; Kyllonen and Christal, 1990; Engle et al., 1999). Some of this work is promising, but in many cases, working memory trainings have been unable to achieve appreciable effect sizes, do not demonstrate sustained and/or transferable effects, and have failed to replicate (Shipstead et al., 2012a,b; Melby-Lervåg and Hulme, 2013; Redick et al., 2013). Indeed, robust meta-analyses have provided strong evidence that past cognitive training efforts—including but not limited to working memory paradigms—do not yield transfer for GCA or its component abilities (Sala and Gobet, 2019).

Although substantial evidence has highlighted the role of working memory in verbal reasoning (Kyllonen and Christal, 1990; Klauer, 1997; Ruff et al., 2003), the lack of successful working memory training effects suggests that targeted training of other cognitive abilities may be worth investigating. Mental modeling is a cognitive ability that draws on working memory (Ruff et al., 2003; Ragni and Knauff, 2013)—as virtually all cognitive abilities do—but has direct, mechanistic ties to spatial cognition and verbal reasoning, and may therefore yield larger effects than efforts to train working memory broadly. Given the evidence for mental modeling as a reasoning-general mechanism, the present study was devised to test whether targeting this specific cognitive ability can produce sustained improvements in reasoning (a domain-general cognitive ability).

If mental modeling is indeed a viable subject of cognitive training, there are important considerations regarding *how* to conduct such a training. Key components of successful cognitive training paradigms include: adaptive training (e.g., attuned to each individual’s performance; Kelly et al., 2014), increases in problem difficulty (Wickens et al., 2013), and performance feedback after each problem (Larson, 1984). For mental models training in particular, one promising direction is to externalize reasoners’ internal mental representations—that is, “build” visible manifestations of the internal spatial representations of complex mental models during the reasoning process. The use of external spatialization tools may afford reasoners better insight into model accuracy through concrete visualization while also reducing burdens on working memory. Informed by educational psychology research, spatial tools allow individuals to better process abstract concepts through concrete visualization, and that can be measured and compared through established methods (Hay et al., 2008). However, it is important that these tools are as simple and color-less as possible, as visual imagery can actually impede the reasoning process (Knauff and Johnson-Laird, 2002). Research on multimedia learning (e.g., translating verbal content into visual images to improve learning) provides support for this notion, as overly complex visual environments during learning can lead to extraneous cognitive processing that distracts from the core processes of the learning paradigm, therefore impeding optical instructional outcomes (Mayer, 2009, 2014; Makransky et al., 2019).

Successful efforts at mental modeling training *via* a simple smartphone application could allow for increased growth in accessibility of such trainings, given the ubiquity of such devices (Poushter, 2016). However, most “brain training” mobile applications are not empirically validated by scientific research before released to the public—and when these apps are scientifically tested, many of them turn out to be completely ineffective at enhancing cognition (Owen et al., 2010; Rabipour and Raz, 2012). This has resulted in a general distrust of “brain training” apps by the scientific community (Simons et al., 2016), as well as legal sanctions against certain apps, such as the FTC’s conviction of Lumosity, for deceptive advertising (Bainbridge and Mayer, 2018).

Therefore, we designed the Mental Models Training App, which requires participants to adaptively complete increasingly difficult reasoning problems while using a spatial modeling tool to construct external mental models. The present study tests whether this app-based training improves verbal deductive reasoning, as measured by the Multidimensional Relational Reasoning Test (MRRT; Cortes et al., 2021). We compared the Mental Models Training App to several control conditions (see Methods) in order to examine which specific components (if any) were causally responsible for improved reasoning performance. Positive effects of the training would provide support for the MMT by demonstrating a causal role of mental modeling ability in verbal deductive reasoning, while also demonstrating the efficacy of a free mobile app that anyone can use to enhance their own reasoning ability. This research is part of a larger effort to translate basic science into applied tools that have the potential to benefit the general public (Wethington and Dunifon, 2012). This study was preregistered on the Open Science Framework.^1^

## 2. Materials and methods

### 2.1. Participants

A total of 382 participants were recruited through Prolific (Palan and Schitter, 2018), and compensated $37.50 for their participation in the full study (i.e., $15 per hour for 2.5 total hours). Participation was limited to adults’ ages 18–35 living in the United States who spoke English as their first language and had not participated in any prior studies from our laboratory. Substantial data removal is standard in online data collection (Buhrmester et al., 2011; Allahbakhsh et al., 2013; Palan and Schitter, 2018), and was anticipated in the present study. We included four attention check items (e.g., please select “True”) throughout the study to screen for participants who were not properly attending to the questions (e.g., rushing through and clicking answers). Thirteen participants were removed for missing a total of two or more attention checks across both sessions, 50 participants were lost due to an error during data collection (sent the wrong survey link), and 18 participants were removed because they did not complete the entire study. Therefore, the final sample included 301 participants (57.8% Female, 38.5% Male, 3.7% Other; mean age = 27.4 years, SD = 7.3; 63.2% Caucasian, 7.3% Asian, 12.6% African American, 5.6% Hispanic; 0% Native American, 11.3% Mixed Race/Other; Total Years of Education: 48.1% 16+ years, 37.5% 13–15 years, 12.9% 12 years, 1.4% 0–11 years; Total Household Income: 19.3% Less than $30,000, 18.3% $30,000–$50,000, 17.9% $50,001–$70,000, 21.6% $70,001–$100,000, 14.3% $100,001–$150,000, 4.3% $150,001–$250,000, 4.3% More than $250,000). All study procedures were approved by the Georgetown University Institutional Review Board, and all participants provided informed written consent before participation.

### 2.2. Study design and procedure

**Figure 1:**
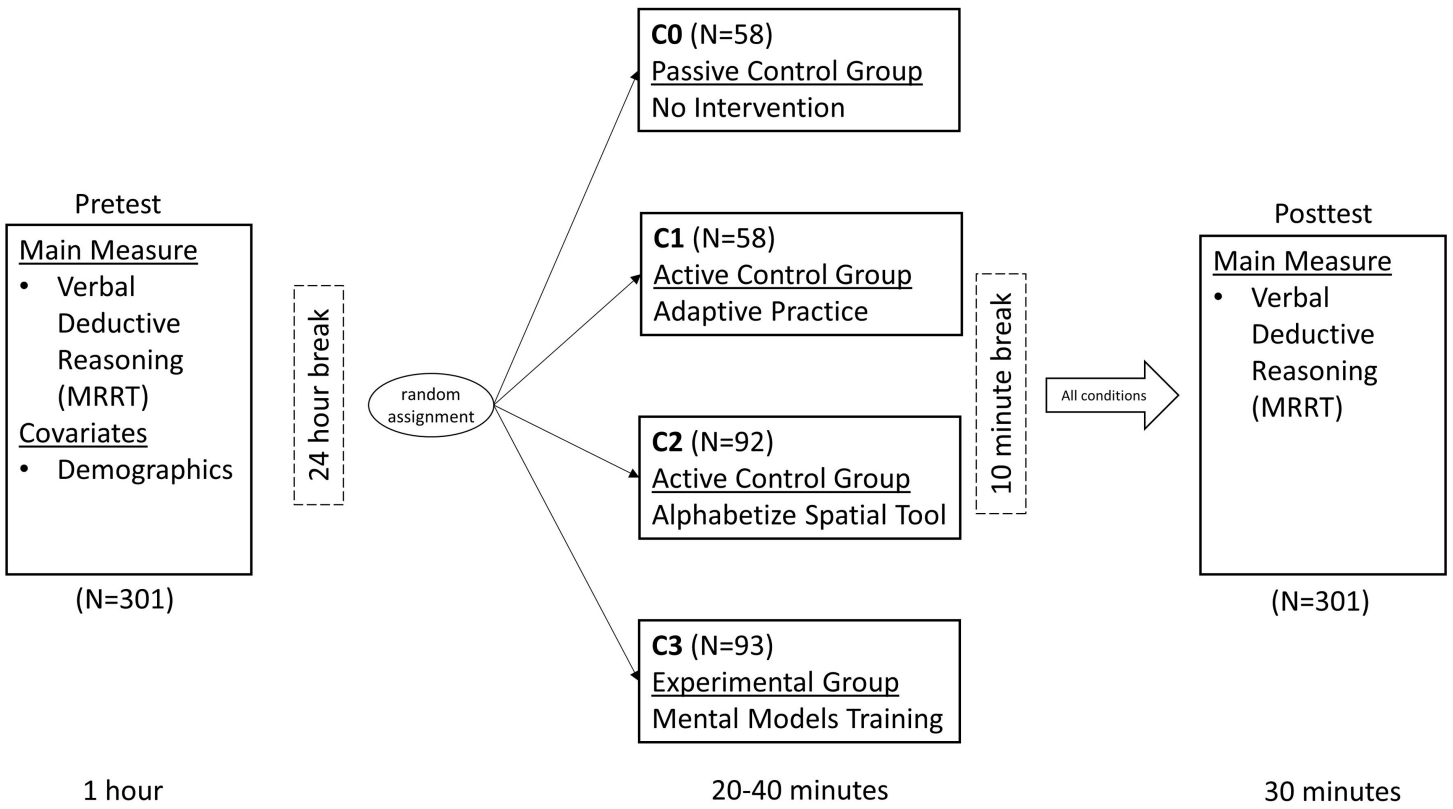
Study design and procedure. Full visual depiction of the study design, cognitive measures administered, sample sizes at each timepoint (for each group), and complete timing information for the length of tasks/interventions administered as well as the break between each session.

**Figure 2:**
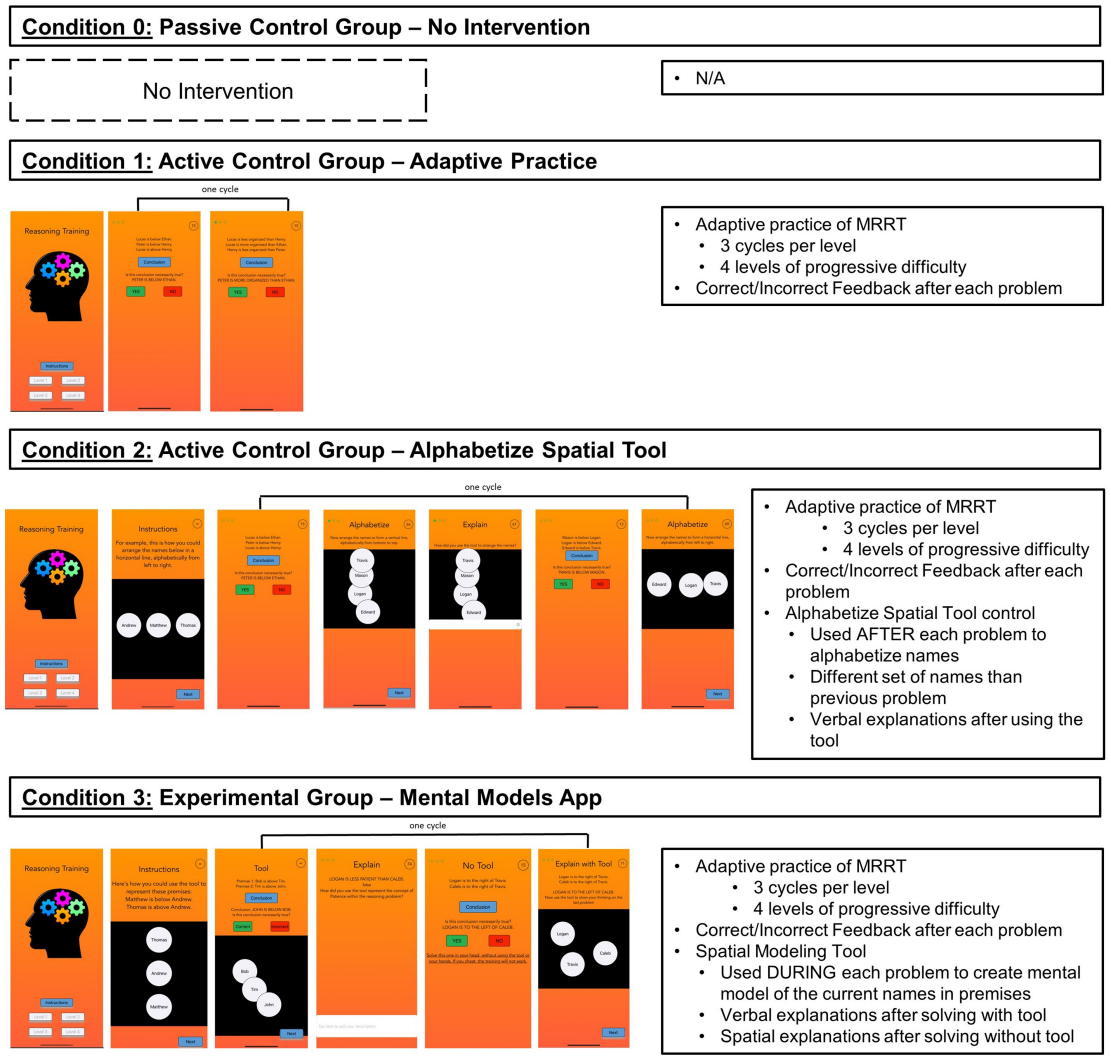
Key components of each condition. Full visual presentation of the app interface for each condition (Left), as well of the key training components of each condition (Right). The app screenshots (Left) represent one cycle from one level, however the design and structure was the same across all 4 levels of the training (as well as each of the 3+ cycles in each level) in each condition. Complete screenshots of the entire instructions section and training levels within each condition can be found at https://osf.io/a8zyn/.

A full visual depiction of the study design and procedure can be found in Figure 1. During the pretest, participants first completed 45 items from the MRRT (Cortes et al., 2021), a measure of verbal deductive reasoning which served as the main outcome measure of the study. After completing the MRRT, participants completed additional measures not analyzed in the present study, with the demographics survey always administered at the end. The entire pretest took approximately 1 h. The following day (24 h later), participants were randomized into one of the four experimental conditions (see *Experimental Conditions* section and Figure 2 for full description of each condition). The timing of the interventions was participant-dependent, as the training application was adaptive to performance in all conditions (except condition 0 in which participants received no intervention), however overall average completion time was approximately 32 min. After completing their respective version of the mobile training application, participants were provided a mandatory 10-min break. Then, all participants completed an appropriately counterbalanced version of the MRRT as a posttest measure of verbal deductive reasoning (to measure change in performance from pretest). The posttest took approximately 30 min. All participants completed the entire study on their iPhones.

### 2.3. Verbal deductive reasoning

**Figure 3:**
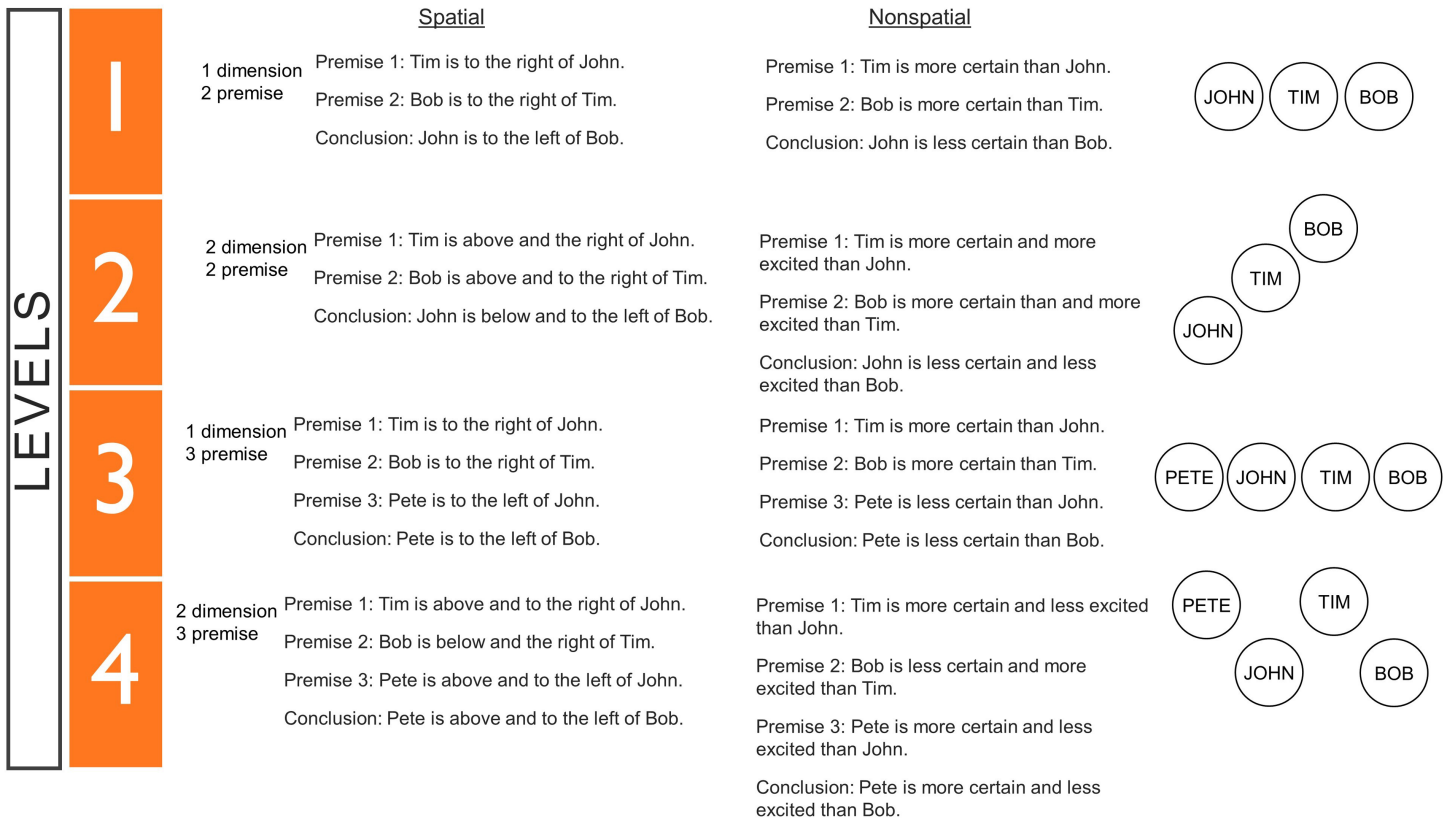
Levels within the mental models training. Full description of the problem types included in each level of the training app in Conditions 1–3. The MRRT problems in these levels were empirically proven to be increasingly difficult (Cortes et al., 2021). The normative average accuracy was 80% for the problems in level 1, 73% for the problems in level 2, 72% for the problems in level 3, and 66% for the problems in level 4.

Verbal deductive reasoning was measured with the MRRT (available for use at https://osf.io/qfvp2/; Cortes et al., 2021). Within each MRRT problem, 2–3 premises and a conclusion were presented (e.g., “Premise 1: Tim is above and the right of John/Premise 2: Bob is above and to the right of Tim/Conclusion: John is below and to the left of Bob”) and participants were instructed to respond with “True” if the conclusion necessarily follows from the premises or “False” if the conclusion could possibly be false (i.e., if it is clearly false from the information in the premises or if the solution is indeterminate). Participants were given up to 90 s to complete each problem and were instructed to solve every problem in their head without the use of pencil/paper or their fingers. The problems in the MRRT were systematically varied along the following stimulus properties: Number of Premises (2 or 3), Number of Dimensions (1 or 2), Relation Type (Spatial or Non-spatial), and Solution (True, False, or Indeterminate). The MRRT was used during pretest, training, and posttest—each implementation contained a different set of names (all two-syllable male names from ranks 50–100 in the list of popular names from the 1990s^2^ in order to prevent participants from seeing repeated problems while preserving (and matching) the underlying stimulus qualities. Two different versions of the MRRT were created (A and B) for the pretest and posttest, both of which contained 45 problems with the same stimulus properties and overall average problem difficulty (72% accuracy), but with different specific names and wording—these versions were counterbalanced across all participants, equally across each of the conditions. For example, half of the participants in each condition completed version A in the pretest and version B in the posttest, while the other half completed version B in the pretest and version A in the posttest. The version of the MRRT in the training was divided into levels based on stimulus properties (number of premises and number of dimensions) which have been empirically proven to impact problem difficulty (for more details, see *Experimental Conditions,* Figure 3, and Cortes et al., 2021). The full stimuli for version A, version B, and the training version of MRRT can be found at https://osf.io/a8zyn/.

### 2.4. Experimental conditions

#### 2.4.1. Condition 0: Passive control group—no intervention

In order to control for the practice effects of completing the MRRT during the pretest and test the effects of each condition against a truly passive control group, Condition 0 was implemented such that participants did not complete any intervention (i.e., they did not download any training app) and simply completed the MRRT posttest 24 h after they completed the pretest.

#### 2.4.2. Similarity across all conditions (excluding Condition 0)

All training conditions (Conditions 1–3) were completed by participants on their iPhones through the TestFlight application, which allowed participants to download a specific version of the training app using a condition-specific password provided by the researchers. Upon opening the app, participants entered their Prolific ID number along with the condition-specific password. The title of the app (“Reasoning Training”), the instructions provided about the reasoning problems (e.g., “Welcome to the Reasoning Training app. This app is designed to help you improve your reasoning skills. The training will get increasingly difficult as you go on, and it is very important that you follow the instructions so that the training is effective.”), and the overall structure of the app (adaptive reasoning training with increasingly difficult problems) was kept the exact same across all conditions (see Figure 2) to create a uniform participant experience and ensure that any group differences were related to specific and intentional differences created between conditions. Within each app, participants were instructed to solve all problems in their head and were given optional 3 min breaks between each level of the training. Participants had 90 total seconds to solve each problem—75 s to view the premises and reason about them, and once participants pressed the “conclusion” button, the conclusion would appear and participants had 15 s to response “Yes” for necessarily true or “No” for not necessarily true. The purpose of this problem timing was to ensure that participants fully solved the problems and processed all of the premise information, rather than focusing solely on the conclusion and using process of elimination. In Condition 3, this ensured that participants fully constructed a mental model before attempting to solve the problem. After each problem, participants received feedback on whether they answered the problem correctly or incorrectly (“Correct” vs. “Incorrect”).

In all training conditions, participants completed the same 4 levels of increasingly difficult MRRT problems (see Figure 3). The verbal deductive reasoning problems in these levels were empirically proven to increasingly difficult based on normative accuracy data (Cortes et al., 2021; Figure 3). Level 1 contained two premise, one dimensional problems with both non-spatial and spatial wording (average accuracy = 80%); Level 2 contained two premise, two dimensional problems with both non-spatial and spatial wording (average accuracy = 73%); Level 3 contained three premise, one dimensional problems with both non-spatial and spatial wording (average accuracy = 72%); and Level 4 contained three premise, two dimensional problems with both non-spatial and spatial wording (average accuracy = 66%). See Figure 3 for full details of each level^3^ for the exact problems within each level. Within each level, participants had to complete 3 successful cycles to advance to the next level. A successful cycle entailed completing two reasoning problems in a row with the correct answer—some of the components within the cycles differed based on condition (see Figure 2 and the Condition 1–3 sections below). After each problem, participants received feedback on whether they answered the problem correctly or incorrectly (“Correct” vs. “Incorrect”). At the end of the app, participants were redirected to a survey which included a mandatory 10-min break, followed by the posttest MRRT. Complete screenshots of the entire instructions section and training levels for each condition (1–3) can be found at https://osf.io/a8zyn/.

#### 2.4.3. Condition 1: Active control group—adaptive practice

In order to control for the effects of practicing verbal deductive reasoning problems in a mobile application, Condition 1 was designed the same as Conditions 2 and 3, except that there was no spatial tool included in the training. Participants still received instructions for solving reasoning problems, the problem timing remained the same, correct/incorrect feedback was still provided after each problem, and the levels still advanced in the same increasingly difficult manner. However, the cycles within each level only included 2 successive reasoning problems (see Figure 2) and there was never any mention or usage of a spatial tool throughout the training.

#### 2.4.4. Condition 2: Active control group—adaptive practice with spatial alphabetization tool

In order to control for the visual, spatial, and motor processes engaged by using a spatial tool during the reasoning training, Condition 2 matched the design of Condition 3, but provided participants with a spatial alphabetization tool (Figure 2) instead of the spatial modeling tool. In the instructions section of the app, participants were introduced to the spatial alphabetization tool and instructed to “arrange the names below in a horizontal line, alphabetically from left to right” (see Figure 2). Participants were instructed to create several different spatial structures throughout the training, depending on the number of names in the premises (e.g., horizontal line, vertical line, triangle, square), and the direction of alphabetization (e.g., left to right, right to left, top to bottom, bottom to top, clockwise, counter clockwise) was evenly distributed across the training.

A key difference from Condition 3 is that, during the levels of the training, participants in Condition 2 were provided with the spatial alphabetization tool *after* each reasoning problem using a different set of names than those shown in the previous problem. This design ensured that participants were not distracted during the reasoning problem (i.e., dividing their attention in counterproductive ways) and that they could not use the alphabetization tool in order to create mental models during the reasoning problems or retrospectively after solving reasoning problems. Relatedly, participants in Condition 2 completed cycles with the following components: 1) complete a reasoning problem without a tool, (2) alphabetize a separate list of names in the specific spatial configuration and alphabetical direction, (3) for non-spatial problems, verbally explain how they used the spatial alphabetization tool to arrange the names to form the alphabetized shape (4) complete a new reasoning problem, (4) alphabetize a separate list of names in the specific spatial configuration and alphabetical direction (see Figure 2). As in all other conditions, participants had to complete 3 successful cycles to advance from one level to the next. At the beginning of each level, participants were shown an example of how the tool could be used to spatially alphabetize the names from the type of problems included in that level (Figure 2).

Typical responses to the verbal explanation prompt for non-spatial problems in Condition 2 included: “I put them alphabetically from left to right,” “I arranged the circles alphabetically from bottom to top in a vertical line,” and “I placed the names alphabetically in a triangle starting lower left and clockwise.” The prevalence of these sorts of responses suggested that the spatial alphabetization tool was generally used as intended. In addition, thorough visual inspection of the alphabetized shapes created throughout the training by participants in this condition confirmed that the alphabetize spatial tool was utilized as intended.

#### 2.4.5. Condition 3: Experimental group—the Mental Models Training App

The defining feature of the Mental Models Training App (Condition 3) was that it provided participants with a spatial modeling tool to create external mental models while solving increasingly difficult reasoning problems in the app’s levels. The spatialization tool was introduced during the instructions section of the app, wherein participants were shown (1) a visual example of how the tool could be used to represent reasoning problems in a spatial manner, (2) how to tap in the workspace to create pre-labeled tokens for each of the names in a reasoning problem, (3) how to move the tokens around within the workspace to create a mental model for a reasoning problem, and (4) an example reasoning problem in which they could use the tool to create a mental model and solve the problem. After completing the instructions, participants began level 1 of the training.

Within each level of the Mental Models Training App (Condition 3), participants completed cycles with the following structure: (1) complete a problem using the spatialization tool to create mental models of the names in the premises, (2) for non-spatial problems, verbally explain how they solved the problem using the spatialization tool, (3) complete a new problem without the use of the spatialization tool, and (4) use the tool to spatially explain how they solved the previous problem (see Figure 2). The goal of this process was to teach participants how to construct mental models externally in a 2-dimensional space and encourage the internalization of this process. As in all other conditions, participants had to complete 3 successful cycles to advance from one level to the next. At the beginning of each level, participants were shown an example mental model for the corresponding type of problems included in that level (Figure 2).

Typical responses to the verbal explanation prompt for non-spatial problems in Condition 3 included: “I used the tool similar to above and below to rank the level of excitement,” “I placed those who were more patient further to the right than those who were less patient,” and “I used the visual tool to show the hierarchy.” The prevalence of these sorts of responses suggested that the spatialization tool was generally used as intended. In addition, thorough visual inspection of the mental models created throughout the training by participants in this condition confirmed that the mental modeling tool was utilized as intended.

### 2.5. Analytic strategy

In order to assess the effects of each training condition on reasoning performance (i.e., MRRT accuracy and RT) from pretest to posttest, we conducted a series of mixed-effects models testing for condition-by-time interactions. Mixed-effects models are appropriate when several repeated measurements or observations (Level 1) are nested within a higher level of data (Level 2; Longford, 1995; Goldstein, 2011). In the present study, stimulus properties of the MRRT (number of dimensions, number of premises, spatial vs. non-spatial wording, true vs. false solution) and timepoint (pretest, posttest) were modeled as a Level 1 variables, and each participant’s demographic variables (age, gender, income, and education) and condition assignment (Condition 0, 1, 2, or 3) were modeled as Level 2 variables. Because we were interested in examining the condition-by-time effects on MRRT accuracy and RT, we performed separate mixed-effects models for these two dependent variables. The condition-by-time effect on accuracy was investigated using a mixed-effects logistic regression because accuracy was a binary variable (i.e., each individual response was either correct or incorrect). RT models were estimated *via* mixed-effects linear regression. All models estimated fixed effects, given that the high number of variables included made random slope estimations computationally infeasible (Bell et al., 2019). All mixed-effects models were fit using the glmer (for accuracy) and lmer (for RT) commands in R Studio (De Boeck et al., 2011; Lee and Grimm, 2018; Verzani, 2014). Significance tests were two-sided.

## 3. Results

### 3.1. Descriptive statistics for pretest variables

Descriptive statics for all variables measured at pretest (separated by condition) can be found in Table 1. Results indicate that all variables were not significantly different across conditions, indicating that each condition contained cognitively and demographically equivalent participants at the start of the experiment (before the various training conditions were administered). This result provides confidence that any training-related effects are likely due to the training conditions rather than extraneous characteristics of the sample in each condition.

**Table 1:** Descriptive statistics for pretest measures across conditions.

	Condition	0	1	2	3	Difference (one-way ANOVA)	
	*N*	58	58	92	93	*F*	*P*
MRRT accuracy	Mean	71%	70%	70%	70%	0.02	0.99
	SD	14%	14%	14%	14%		
MRRT RT (seconds)	Mean	31.19	33.61	31.91	33.02	0.44	0.72
	SD	13.82	17.06	10.60	11.99		
Age	Mean	27.03	26.17	27.57	28.18	0.96	0.41
	SD	4.71	5.42	4.78	10.91		
Gender	Female	60%	53%	61%	56%	0.36	0.78
	Male	36%	43%	34%	42%		
	Other	4%	4%	5%	2%		
Income bracket	Less than $30,000	26%	16%	16%	20%	0.18	0.91
	$30,000–$50,000	16%	19%	21%	17%		
	$50,001–$70,000	16%	19%	18%	18%		
	$70,001–$100,000	22%	28%	21%	18%		
	$100,001–$150,000	14%	16%	13%	15%		
	$150,001–$250,000	3%	2%	4%	6%		
	More than $250,000	3%	2%	7%	4%		
Total years of education	0–11	2%	2%	1%	1%	0.13	0.95
	12	12%	12%	12%	15%		
	13–15	36%	38%	40%	35%		
	16+	50%	48%	47%	48%		

Condition 0, no intervention; Condition 1, adaptive practice; Condition 2, alphabetize spatial tool; Condition 3, mental models training.

### 3.2. Effects of training conditions on reasoning performance

**Figure 4:**
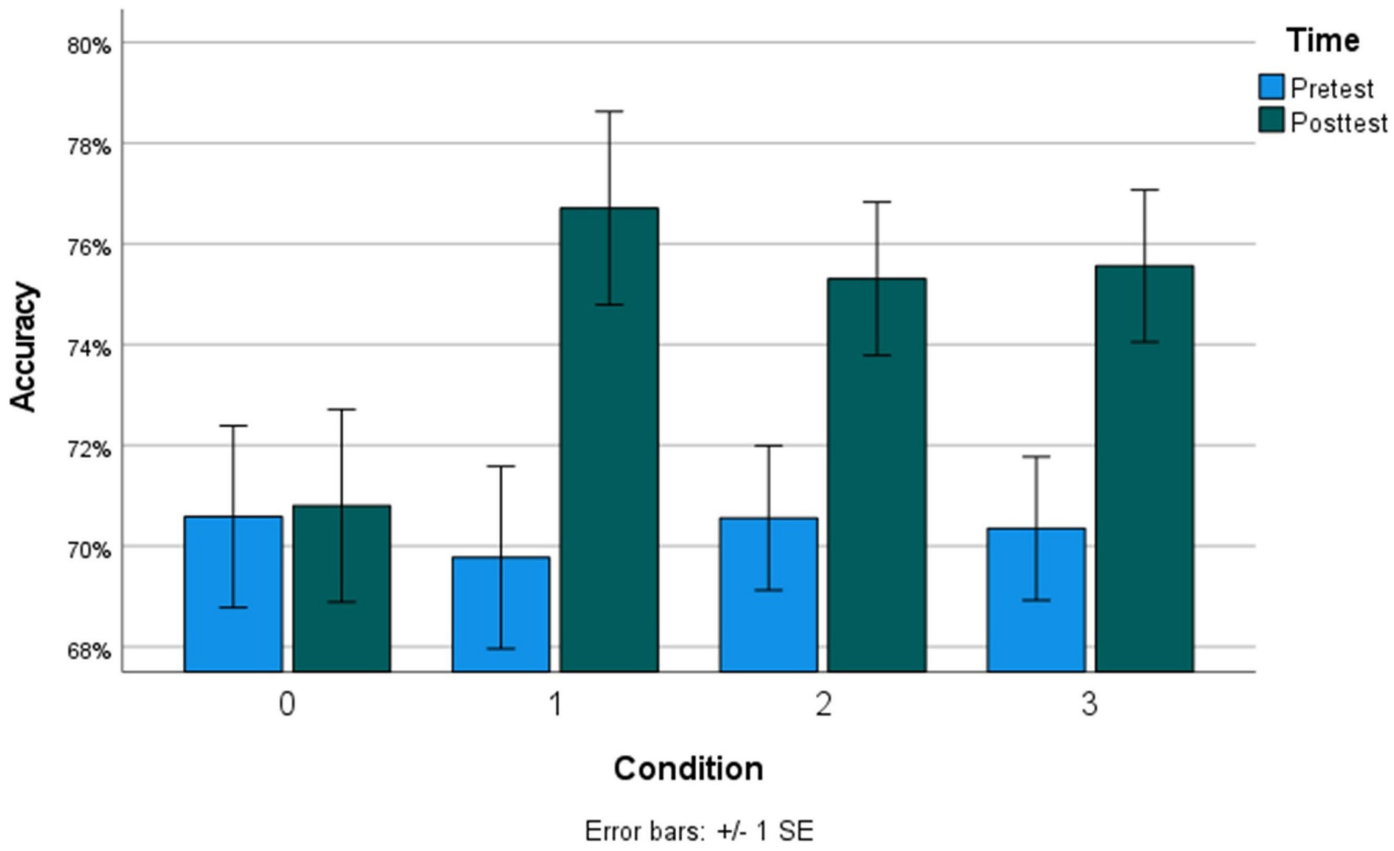
Mean accuracy at each timepoint across all conditions. Condition 0, no intervention; Condition 1, adaptive practice; Condition 2, alphabetize spatial tool; Condition 3, mental models training.

**Figure 5:**
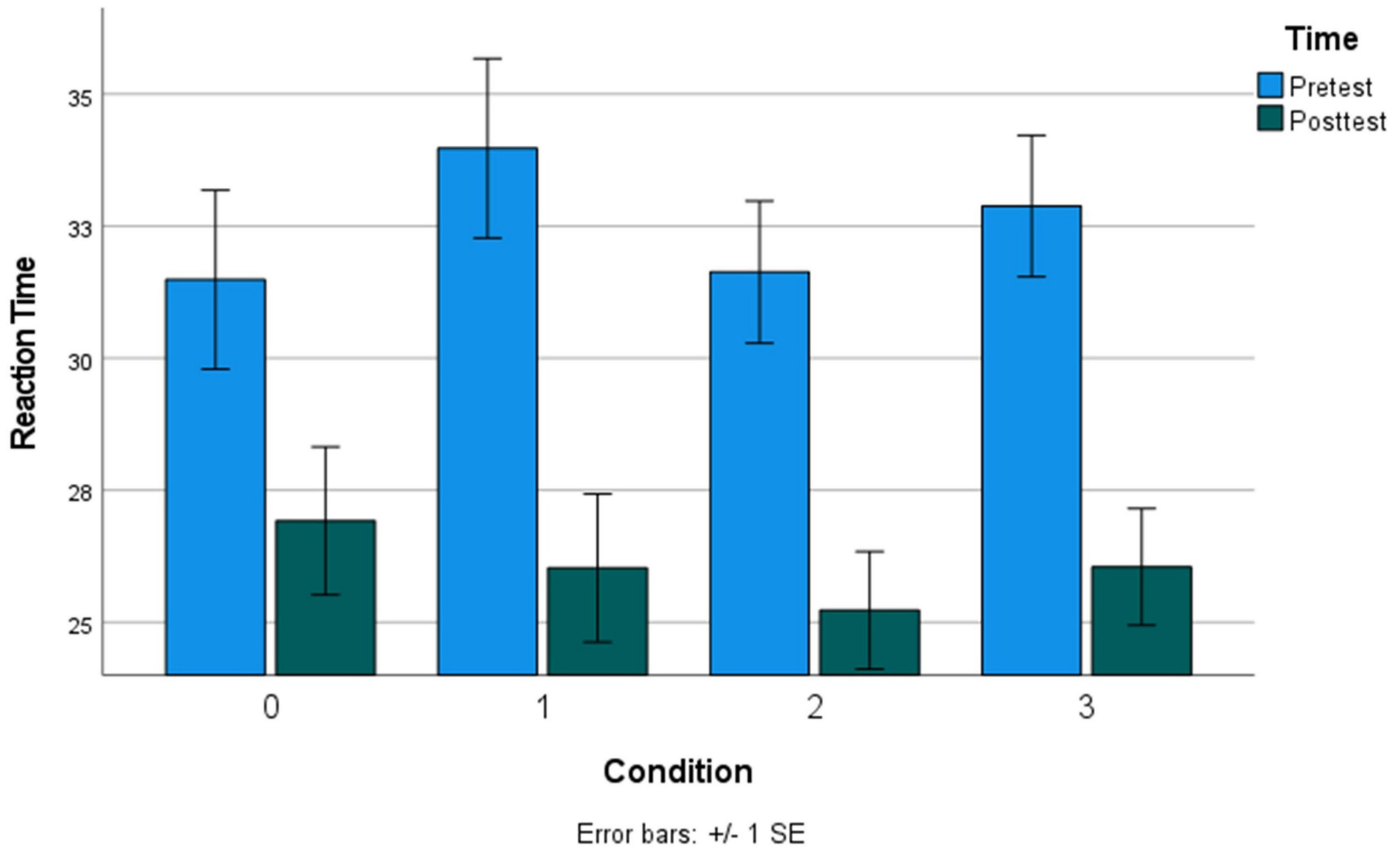
Mean reaction time (seconds) at each timepoint across all conditions. Condition 0, no intervention; Condition 1, adaptive practice; Condition 2, alphabetize spatial tool; Condition 3, mental models training.

We ran two mixed-effects models (Model 1: Accuracy, mixed-effects logistic regression; Model 2: RT, mixed-effects linear regression) to examine whether each of the training conditions (1–3) significantly improved MRRT performance from pretest to posttest, using the passive control condition with no intervention (condition 0) as the reference factor level. All models controlled for stimulus properties of the MRRT problems (relation type, premises, dimensions, and solution) and demographic characteristics of the participants (Age, Gender, Income Bracket, and Total Education). Results indicated significant condition-by-time effects of all three conditions (1–3) on MRRT accuracy (Table 2) and RT (Table 3). Condition 1 (adaptive practice) showed the largest training effects compared to condition 0 (passive control), as participants in condition 1 were 1.46 times more likely to provide the correct response in 3.26 fewer seconds. Participants in condition 2 (alphabetize spatial tool) were 1.31 times more likely to provide the correct response in 1.98 fewer seconds when compared to condition 0 (passive control). Participants in condition 3 (mental models training) were 1.35 times more likely to provide the correct response in 2.22 fewer seconds. Bar graphs of the mean accuracy and RT for each condition at each timepoint can be found in Figures 4, 5, respectively. Additional models comparing the effects between the training app conditions (condition 3 vs. condition 1, condition 2 vs. condition 1, condition 3 vs. condition 2) revealed no significant differences in the size of the training effects between conditions 1 and 3 on accuracy (all *p* > 0.38) or RT (all *p* > 0.07).

**Table 2:** Mixed-effects logistic regression model for condition-by-time effects on accuracy (fixed effects).

**Accuracy**			
Predictors	Odds ratios	Confidence interval	*P*
(Intercept)	2.17	1.23–3.83	**0.008**
Condition [1]	0.66	0.45–0.97	**0.035**
Condition [2]	0.74	0.52–1.04	0.085
Condition [3]	0.71	0.50–1.00	**0.048**
Timepoint	1.00	0.88–1.14	0.956
Relation type [Spatial]	1.09	1.03–1.15	**0.002**
Premises [2 Premise]	1.54	1.46–1.63	**<0.001**
Dimensions [1 Dimension]	1.41	1.37–1.45	**<0.001**
Solution [Indeterminate]	0.76	0.71–0.81	**<0.001**
Solution [True]	1.10	1.03–1.18	**0.006**
Age	1.00	0.99–1.01	0.806
Gender [Male]	1.14	0.96–1.36	0.128
Income bracket	1.07	1.03–1.12	**0.002**
Total education	1.08	0.93–1.26	0.299
Condition [1] * Timepoint	1.46	1.12–1.74	**<0.001**
Condition [2] * Timepoint	1.31	1.11–1.53	**0.001**
Condition [3] * Timepoint	1.35	1.11–1.59	**<0.001**
**Random effects**			
σ^2^	3.29		
τ_00 ID_	0.51		
ICC	0.13		
N_ID_	301		
Observations	27,563		
Marginal R^2^/Conditional R^2^	0.046/0.173		

Condition 1, adaptive practice; Condition 2, alphabetize spatial tool; Condition 3, mental models training. Condition 0 (no intervention) was the reference level in this model: The following variables were dummy coded. Relation Type: spatial vs. non-spatial; Premises: two premise vs. three premise; Dimensions: one-dimension relations vs. two-dimension relations; Solution: False vs. Indeterminate and False vs. True; Gender: 0, female; 1, male. Bold values indicate significant effects (*p* < 0.05).

**Table 3:** Mixed-effects linear regression model for condition-by-time effects on RT (fixed effects).

**Reaction time**				
Predictors	Estimates	Confidence interval		*P*
(Intercept)	37.91	29.97–45.84		**<0.001**
Condition [1]	5.72	1.26–10.17		**0.012**
Condition [2]	2.72	−1.30–6.74		0.085
Condition [3]	3.90	−0.11–7.92		0.057
Timepoint	−4.51	−5.40 to −3.62		**<0.001**
Relation type [Spatial]	−2.24	−2.63 to −1.86		**<0.001**
Premises [2 Premises]	−7.97	−8.35 to −7.58		**<0.001**
Dimensions [1 Dimension]	−6.40	−5.99 to −6.81		**<0.001**
Solution [Indeterminate]	0.56	0.09–1.04		**0.021**
Solution [True]	−1.06	−1.53 to −0.58		**<0.001**
Age	0.15	−0.02–0.32		0.083
Gender [Male]	2.56	0.02–5.11		**0.049**
Income bracket	−0.54	−1.14–0.07		0.084
Total education	−1.97	−4.18–0.25		0.082
Condition [1] * Timepoint	−3.28	−4.53 to −2.02		**<0.001**
Condition [2] * Timepoint	−1.98	−3.11 to −0.84		**0.001**
Condition [3] * Timepoint	−2.22	−3.35 to −1.09		**<0.001**
**Random effects**				
σ^2^	269.94			
τ_00 ID_	119.57			
ICC	0.31			
N_ID_	301			
Observations	27,563			
Marginal R^2^/Conditional R^2^	0.099/0.375			

Condition 1, adaptive practice; Condition 2, alphabetize spatial tool; Condition 3, mental models training. Condition 0 (no intervention) was the reference level in this model. The following variables were dummy coded. Relation Type: spatial vs. non-spatial; Premises: two premise vs. three premise; Dimensions: one-dimension relations vs. two-dimension relations; Solution: False vs. Indeterminate and False vs. True; Gender: 0, female; 1, male. Bold values indicate significant effects (*p* < 0.05).

### 3.3. Within-training differences between conditions

Next, we examined differences in performance within the training app across conditions 1–3 (condition 0 was not included as it did not include the app intervention). Participants in condition 1 (adaptive training) completed the training in an average of 21.93 min, which was significantly shorter (about half as long) than the average completion time in condition 2 (alphabetize spatial tool; 38.43 min) and condition 3 (mental models training; 37.55 min; Table 4). This was not surprising given that condition 1 contained half as many training components as conditions 2 and 3 (see Figure 2). For this reason, the remaining analyses of within-training focus on the number of problems completed within the training levels, which directly tracks with the number of cycles participants had to successfully complete before advancing to the following level (i.e., how well they were performing within each level).

**Table 4:** Total training time and number of problems completed during the app training across conditions 1–3.

	Condition	1	2	3	One-way ANOVA	
	*N*	58	92	93	*F*	*P*
Total Training Time (minutes)	Mean	21.93	38.43	37.55	**27.61**	**<0.001**
	SD	12.02	15.79	14.19		
Total number of problems completed in training	Mean	46.41	46.96	42.14	2.17	0.12
	SD	17.29	18.05	14.54		
Level 1 number of problems	Mean	9.51	9.69	8.97	0.65	0.52
	SD	5.73	4.25	3.31		
Level 2 number of problems	Mean	11.28	12.87	12.27	1.09	0.34
	SD	6.26	6.55	6.41		
Level 3 number of problems	Mean	12.21	11.07	8.47	**9.98**	**<0.001**
	SD	6.56	5.96	3.41		
Level 4 number of problems	Mean	13.41	13.33	12.43	2.17	0.12
	SD	5.58	7.73	7.92		

Condition 1, adaptive practice; Condition 2, alphabetize spatial tool; Condition 3, mental models training. Condition 0 (no intervention) was not included in this table because participants in that condition did not complete any form of app training. Bold values indicate significant effects (*p* < 0.05).

**Figure 6:**
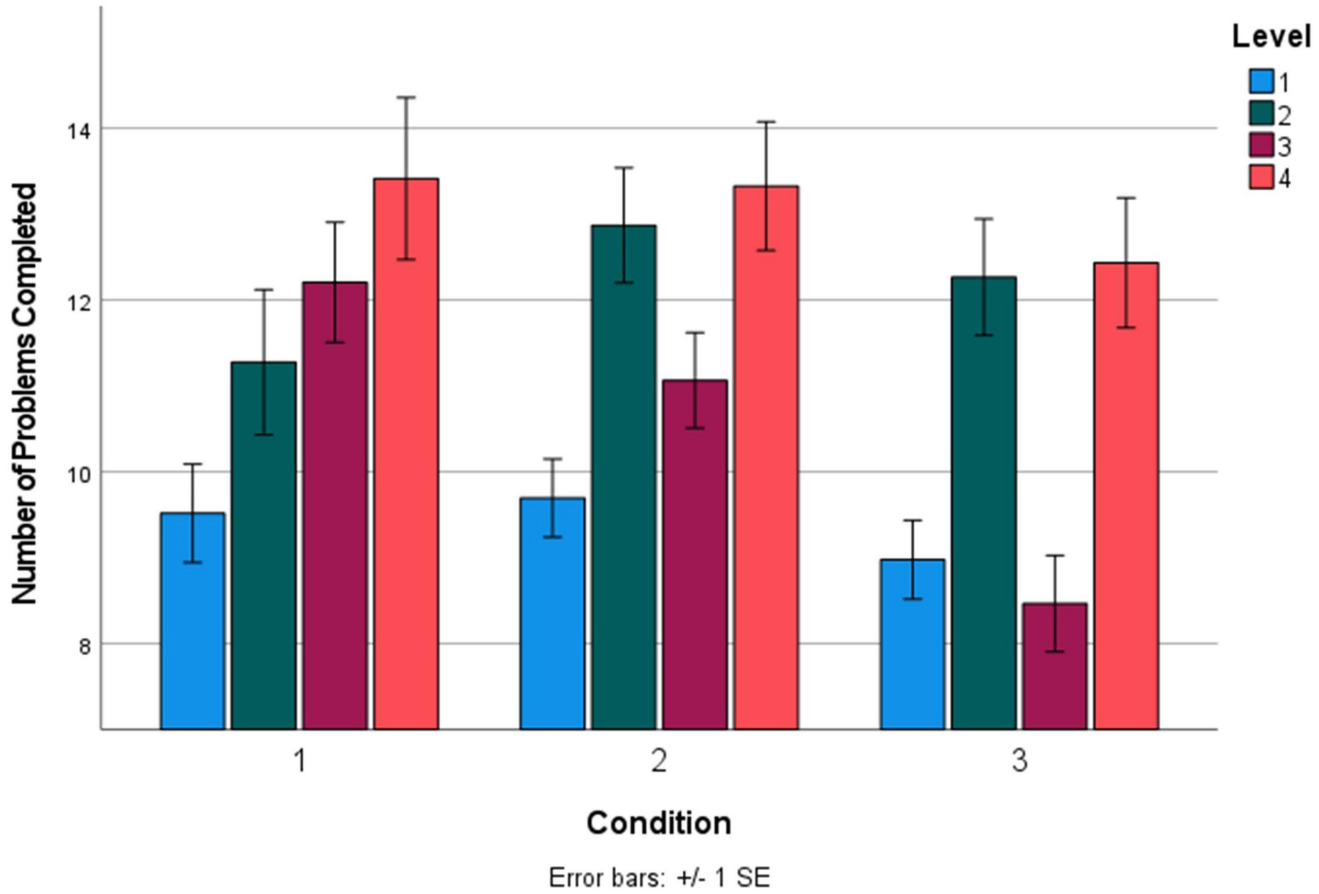
Mean number of problems completed in each level of the training across conditions 1–3. Condition 1, adaptive practice; Condition 2, alphabetize spatial tool; Condition 3, mental models training.

The total number of reasoning problems completed in the training was not significantly different across conditions (Table 4). However, in level 3 of the training, participants in condition 3 (mental models training) completed significantly fewer problems (mean of 8.47 problems, or 4.1 successful cycles) than both condition 2 (alphabetize spatial tool; mean of 11.07 problems, or 5.53 successful cycles) and condition 1 (adaptive practice; mean of 12.21 problems, or 6.11 successful cycles; Table 4). Completing fewer problems indicated improved performance within a training level, as 3 successful cycles (one successful cycle included two subsequent correct reasoning problems) were required to advance from each level—the higher number of problems completed within a level, the more problems a participant answered incorrectly. In sum, participants in the Mental Models Training App condition answered fewer problems incorrectly (i.e., performed better) in level 3 compared to the active control conditions. Level 3 problems contained three premise, one-dimension reasoning problems. There were not significant differences in number of problems completed in any other levels (Table 4), though the differences in progression through the training can be visualized in Figure 6, which contains a bar graph representing the mean number of problems completed during the training across conditions 1–3.

### 3.4. Exploratory analyses

Based on the finding that participants in Condition 3 showed improved performance on 3-premise problems in level 3 of the mobile training app (compared to Conditions 1 and 2), we conducted exploratory analyses testing for a significant three-way interaction between Condition-Time-Premises on reasoning performance (examining the posttest training effects in Condition 3 as compared to the other conditions). Results indicated no significant Condition-Time-Premises interaction for Condition 3 compared to: Condition 0 (Accuracy: Odds Ratio = 1.08, *CI* = 0.66–1.27, *p* = 0.602; RT: Estimated effect: −0.30 s, *CI =* −1.96-2.56, *p* = 0.795), Condition 1 (Accuracy: Odds Ratio = 1.17, *CI* = 0.60–1.15, *p* = 0.262; RT: Estimated effect: 0.65 s, *CI =* −2.90-1.60, *p* = 0.572), or Condition 2 (Accuracy: Odds Ratio = 0.92, *CI* = 0.81–1.44, *p* = 0.593; RT: Estimated effect: 0.24 s, *CI =* −2.21-1.73, *p* = 0.813).

## 4. Discussion

The present study provides empirical evidence that a mental model-based cognitive training mobile application (“The Mental Models Training App”) significantly improved verbal deductive reasoning performance, as indicated by increased accuracy and reduced reaction time on the MRRT (Cortes et al., 2021), compared to a passive control group which received no intervention. However, contrary to our preregistered hypotheses, the training-induced improvements in the Mental Models Training App condition were not significantly different than the improvements in both of the active control conditions of the app intervention—one which included adaptive practice of the MRRT (condition 1), and the other which included adaptive practice as well as an alphabetize spatial tool control task (condition 2). Specifically, the adaptive practice training (condition 1) led to the nominally highest improvements in reasoning performance, despite taking roughly half the amount of time (~22 min) as the mental models training and the alphabetize spatial tool control training (~38 amounts). These results demonstrate that simply practicing reasoning problems within any version of the mobile app led to improved reasoning performance immediately after completing the training.

We did not find evidence for an additive benefit of the spatialization tool, nor a closely matched control version of that tool, for improving reasoning performance after the training. In line with prior research on cognitive training (Schubert et al., 2014), it is possible that the practice-based training (in the adaptive practice condition) may be more effective than strategy-based training (in the mental models conditioning) at improving reasoning performance in the short-term (i.e., after one session). Relatedly, the additional cognitive demands of the mental models training (i.e., creating visualizations of mental models in-between and during trials) may have produced fatigue effects which were not present in the adaptive training condition (which took half the time to complete and did not involve any sort of multi-tasking between problems). Future research should examine the long-term effects of repeated usage of the Mental Models Training App, as it is possible that if the intervention was completed multiple times across several weeks, and posttest performance was measured on the scale of months rather than minutes, the Mental Models Training App may be the most effective at promoting long-term retention of improvements and overall strategy changes compared to basic practice in the control condition. Therefore, while the present results demonstrate the ability of the Mental Models Training App to enhance verbal reasoning, they do not support the mental models theory-based hypothesis that directly training participants’ mental modeling ability yields improved performance beyond the effects of adaptive, increasingly difficult practice of reasoning problems in a cognitive training mobile application.

However, we did find evidence that the spatial modeling tool directly improved performance *during* the mobile training app. Specifically, participants in the Mental Models Training App training completed level 3 of the training (one-dimension, three premise problems) with significantly fewer total attempts (an average of 8 problems completed compared to 12 problems in both of the control conditions). Previous research on deductive verbal reasoning has found that the single most impactful stimulus factor on problem difficulty is the number of premises (Cortes et al., 2021). In particular, the increase from two premises to three premises results in a 10% reduction in accuracy (Cortes et al., 2021), due to the additional demands a third premise places on working memory (Klauer, 1997; Johnson-Laird, 2001; Goodwin and Johnson-Laird, 2005). In the present data, access to the spatial modeling tool during the training completely wiped out this effect on difficulty (0% change in difficulty compared to 10% in prior data; see Figure 6), indicating that externalizing mental models improved adaptation when reasoning becomes more difficult, perhaps by reducing working memory load during reasoning. However, it should be noted that this within-training improvement on three premise problems did not transfer to posttest reasoning performance.

Future research should test for transfer effects of the Mental Models Training App to other kinds of reasoning, such as causal (Waldmann and Hagmayer, 2013; Khemlani et al., 2014), temporal (Kelly et al., 2020), categorical (Copeland, 2006), and visuospatial reasoning (Elliott and Tyler, 1986; Waschl et al., 2017), all of which are theorized to be supported by the mental modeling resource (Johnson-Laird, 1980, 2004, 2010; Goel et al., 2000; Khemlani and Johnson-Laird, 2012; Ragni and Knauff, 2013; Khemlani et al., 2014; Johnson-Laird et al., 2017). Moreover, research should examine the effects of the intervention on different age groups, such as older adults where cognitive training has yielded the most substantial benefits (Willis et al., 2006; Kueider et al., 2012), or younger children where milestones along their developmental cascade are significantly predictive of future cognitive abilities (Piaget, 1952; Gibson, 1988; Bornstein et al., 2013; Adolph and Tamis-LeMonda, 2014; Libertus et al., 2016). Given recent evidence demonstrating transfer effects from spatially enriched education to verbal deductive reasoning (Cortes et al., 2021), it is possible that an intervention which directly trains spatial scanning ability, a core spatial cognitive process known to support reasoning (Knauff, 2009), may be more effective at producing post-training reasoning performance enhancements than an intervention which directly training participants’ reasoning (such as the Mental Models Training App). Future research should compare the effects of spatial and reasoning training on posttest reasoning performance within the same sample.

Finally, we present the Mental Models Training App as a free mobile application (available on the Apple App store^4^), in the hope that it may be useful for individuals seeking to improve their reasoning ability.

## Data availability statement

The datasets presented in this study can be found in online repositories. The names of the repository/repositories and accession number(s) can be found at: The data and code for this study can be found in the Open Science Framework (https://osf.io/a8zyn/).

## Ethics statement

The studies involving human participants were reviewed and approved by Georgetown University Institutional Review Board. The patients/participants provided their written informed consent to participate in this study.

## Author contributions

AG, RC, and AW: conceptualization and writing—review and editing. RC: methodology, formal analysis, investigation, data curation, visualization, project administration, and funding acquisition. RC and AW: writing—original draft preparation. AG: supervision. All authors contributed to the article and approved the submitted version.

## Funding

This research was supported by grants to AG from the National Science Foundation (DRL-1420481, EHR-1661065, and EHR-1920682) and the John Temple Foundation for AG and AW [ID 61114]. RC was supported by a National Science Foundation Graduate Research Fellowship and by the Patrick Healy Graduate Fellowship from Georgetown University.

## Conflict of interest

RC and AG are the developers of intellectual property owned by Georgetown University related to the Mental Models Training App technology that is described in this manuscript. Although the app is free to download, it includes advertisements and in-app purchases that have the potential to generate revenue for RC, AG, and Georgetown University. Google manages all aspects of the advertisements including content and placement. Furthermore, in-app advertisements do not necessarily represent the data presented in the app.

The remaining author declares that the research was conducted in the absence of any commercial or financial relationships that could be construed as a potential conflict of interest.

## Publisher’s note

All claims expressed in this article are solely those of the authors and do not necessarily represent those of their affiliated organizations, or those of the publisher, the editors and the reviewers. Any product that may be evaluated in this article, or claim that may be made by its manufacturer, is not guaranteed or endorsed by the publisher.

## Footnotes

^1^ https://osf.io/4b7kn

^2^ https://www.ssa.gov

^3^ https://osf.io/a8zyn

^4^ https://apps.apple.com/us/app/mental-models-training/id1664939931
